# Enhanced deep learning model enables accurate alignment measurement across diverse institutional imaging protocols

**DOI:** 10.1186/s43019-023-00209-y

**Published:** 2024-01-12

**Authors:** Sung Eun Kim, Jun Woo Nam, Joong Il Kim, Jong-Keun Kim, Du Hyun Ro

**Affiliations:** 1 Department of Orthopaedic Surgery, Seoul National University College of Medicine, 101 Daehak-ro, Jongno-gu, Seoul, 110-744 Republic of Korea; 2 Department of Orthopaedic Surgery, Seoul National University Hospital, Seoul, South Korea; 3 CONNECTEVE Co., Ltd, Seoul, South Korea; 4 Department of Orthopaedic Surgery, Kangnam Sacred Heart Hospital, Hallym University College of Medicine, Seoul, South Korea; 5 Department of Orthopaedic Surgery, Heung-K Hospital, Gyeonggi-do, South Korea

**Keywords:** Deep learning, Full-leg radiographs, Alignment measurement, Imaging protocol, Accuracy

## Abstract

**Background:**

Achieving consistent accuracy in radiographic measurements across different equipment and protocols is challenging. This study evaluates an advanced deep learning (DL) model, building upon a precursor, for its proficiency in generating uniform and precise alignment measurements in full-leg radiographs irrespective of institutional imaging differences.

**Methods:**

The enhanced DL model was trained on over 10,000 radiographs. Utilizing a segmented approach, it separately identified and evaluated regions of interest (ROIs) for the hip, knee, and ankle, subsequently integrating these regions. For external validation, 300 datasets from three distinct institutes with varied imaging protocols and equipment were employed. The study measured seven radiologic parameters: hip-knee-ankle angle, lateral distal femoral angle, medial proximal tibial angle, joint line convergence angle, weight-bearing line ratio, joint line obliquity angle, and lateral distal tibial angle. Measurements by the model were compared with an orthopedic specialist's evaluations using inter-observer and intra-observer intraclass correlation coefficients (ICCs). Additionally, the absolute error percentage in alignment measurements was assessed, and the processing duration for radiograph evaluation was recorded.

**Results:**

The DL model exhibited excellent performance, achieving an inter-observer ICC between 0.936 and 0.997, on par with an orthopedic specialist, and an intra-observer ICC of 1.000. The model's consistency was robust across different institutional imaging protocols. Its accuracy was particularly notable in measuring the hip-knee-ankle angle, with no instances of absolute error exceeding 1.5 degrees. The enhanced model significantly improved processing speed, reducing the time by 30-fold from an initial 10–11 s to 300 ms.

**Conclusions:**

The enhanced DL model demonstrated its ability for accurate, rapid alignment measurements in full-leg radiographs, regardless of protocol variations, signifying its potential for broad clinical and research applicability.

## Background

Accurate identification of anatomic landmarks is essential for understanding patient anatomy, assessing pathological conditions and planning treatment. The full-leg plain radiograph, which encompasses the hip, knee and ankle, provides valuable information regarding the lower extremities [1]. This radiograph is not only provides information on knee alignment but also limb length discrepancies, osteoarthritis severity, and pre- and postoperative assessment [2]. Conventional measurement methods using rulers or digital calipers, require significant practitioner expertise. To overcome this limitation, deep learning (DL) algorithms have emerged [3]. However, despite the high performance of existing DL models, models that can measure a wide range of knee parameters comprehensively are lacking in number, and studies featuring such extensive capabilities often lack external validation [4–9].

In an earlier study, the authors developed a DL model for detecting anatomic landmarks in full-leg plain radiographs [7]. With a processing time ranging from 10 to 11 s, the model could measure radiologic parameters such as the hip-knee-ankle angle (HKAA), medial proximal tibial angle (MPTA), lateral distal femoral angle (LDFA) and joint line convergence angle (JLCA). The inter-observer reliability was comparable to an orthopedic specialist, and the model showed outstanding reproducibility [7].

However, our initial results were derived solely from data of a single institution. Considering the variability in X-ray equipment and protocols among different hospitals, it was essential to investigate the model's universal applicability. In addition, we expanded the model's scope to encompass additional radiologic measurements routinely employed in clinical practice. Furthermore, there was a need to shorten the processing time, particularly when handling extensive datasets of radiographs for evaluation.

Therefore, the primary aims of this study are two-fold: first, to validate our upgraded DL model's applicability in detecting anatomic landmarks on full-leg plain radiographs from multiple healthcare institutions; second, to assess its accuracy in the context of an extended array of radiological measurements. As a secondary aim, we sought to compare the processing speed of this enhanced model with that of its predecessor.

## Methods

### Training the DL model

#### Study subjects

This study was approved by the Institutional Review Board, and the requirement for informed consent was waived due to the study's retrospective design. We used the same training set as in our previous study [7]. A total of 13,192 patients who underwent full-leg knee radiographs between January 2009 and December 2019 in a single tertiary hospital were initially included. Poor-quality radiographs (e.g. suboptimal contrast, improper positioning, and shape distortion) and those with missing or ambiguous anatomical landmarks (e.g. incomplete coverage from the femur head to the tibia plafond or difficulty in pinpointing specific landmarks) were excluded (n = 1,980). Finally, right-sided radiographs from 11,212 patients were used for model training.

#### Data acquisition and processing

Radiographs were obtained under controlled lower-extremity rotation to standardize the standing position (patella facing-forward). These images were retrieved from the institute’s Picture Archiving and Communication System (PACS). For patients with multiple full-leg radiographs, a single image was randomly chosen. Ground truth masks, proportional to the input image dimensions, were generated by annotating each anatomical landmark and were subsequently incorporated into the model.

#### Anatomical landmarks annotation and angle measurement

**Fig. 1:**
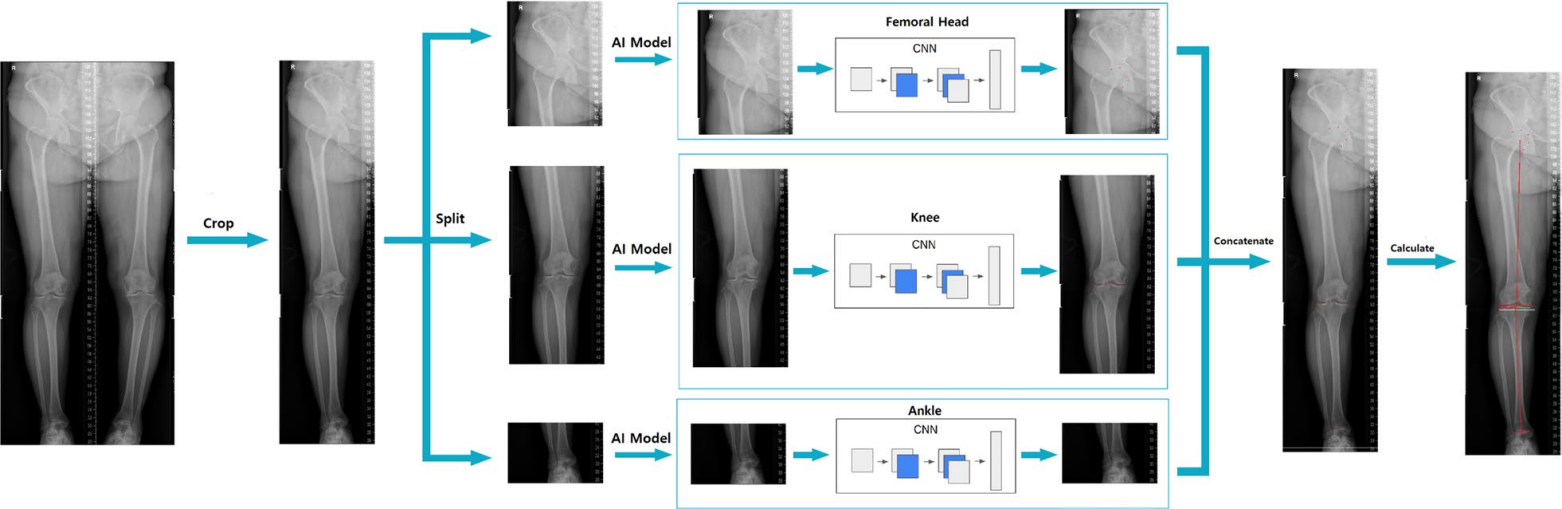
Schematic process of training the DL model. CNN; convolutional neural network

Two orthopedic surgeons annotated 19 anatomical landmarks for training the DL model. The landmarks included five points on the circumference of the femoral head (used to infer its center), the medial and lateral distal points of the femur, the intercondylar fossa, the medial and lateral tibial articular edges, the medial and lateral spines of the tibia, the intercondylar eminence, the midpoints of the medial and lateral tibial plateau, the medial and lateral edges of the talar dome, the center of the talar dome, and the tip of the fibula head. (Fig. 1).

Parameters derived from these landmarks included HKAA, LDFA, MPTA, JLCA, weight-bearing line ratio (WBL ratio), joint line orientation angle (JLOA), and lateral distal tibial angle (LDTA). The HKAA represents the angle formed by connecting the centers of the femoral head, knee, and ankle [10]. The LDFA is the angle between the mechanical axis of the femur and the distal joint line of the femur [10]. The MPTA is the angle between the mechanical axis of the tibia and the proximal joint line of the tibia [10]. The JLCA is the angle between the distal joint line of the femur and the proximal joint line of the tibia [11]. The WBL ratio indicates the position of the weight loading on the knee in X-rays [12]. The JLOA is the angle between the knee joint line and the ground [13]. The LDTA is the angle formed by the mechanical axis of the tibia and the joint line of the distal tibia [14].

#### Model implementation

The full-leg radiographs were divided into three anatomical regions: femoral head, knee, and ankle, based on a rule-based partitioning system. The upper quarter of each image was allocated to the femoral head, the middle half to the knee, and the lower quarter to the ankle (Fig. 1). Individual models were trained for these regions, incorporating techniques such as color jittering, normalization, and image augmentations [15].

The convolutional neural network (CNN) was utilized for training. For images with extremely high resolutions causing substantial data size variations, a preprocessing step scaled the data size down by 90%. To optimize the model's speed and precision, we integrated the Disentangled Keypoint Regression (DEKR) training methodology. Originally devised for human pose estimation, DEKR employs a bottom-up approach, enhancing the quality of keypoint localization and achieving satisfactory pose estimation results [16]. During training, several hyperparameters including learning rate, input size, and optimizer settings were optimized. The model employed various loss functions, including Offset Loss, Heatmap Loss, Adaptive Wing Loss, and a Custom Loss function, each fine-tuned for specific tasks.

Adaptive Wing Loss, often used in facial landmark detection, addressed limitations of Mean Squared Error (MSE) loss in Heatmap regression. MSE tends to blur predictions and gives equal weight to all pixels, including background pixels, which may lead to inaccuracies [17]. To overcome these shortcomings, we utilized the advantages of Adaptive Wing Loss, whose characteristics align well with Heatmap regression tasks. Additionally, a Custom Loss function was developed to penalize significant discrepancies between predicted and actual landmarks, thus adding a layer of complexity to the training but improving model performance and accuracy.

### Validation and performance evaluation

For validation, a separate set of 300 full-leg radiographs was annotated by an orthopedic specialist, with over seven years of expertise in the field. Out of these radiographs, 100 radiographs were sourced using the identical X-ray equipment that was deployed for the training set, and these were designated as originating from Institute A. In contrast, the subsequent 200 radiographs were acquired from two different institutions (with 100 from each), labeled as Institute B and C. These 200 radiographs were captured with X-ray equipment that differed from the one used in our training set. To ensure a representative sample, these 300 radiographs were randomly selected from the institutes. Importantly, this selection process did not exclude radiographs showing severe joint space narrowing or sclerotic changes due to osteoarthritis, nor did it exclude images with pronounced varus-valgus or flexion–extension deformities. This was intentional to assess the model’s ability to accurately measure under varied pathological conditions.

**Fig. 2:**
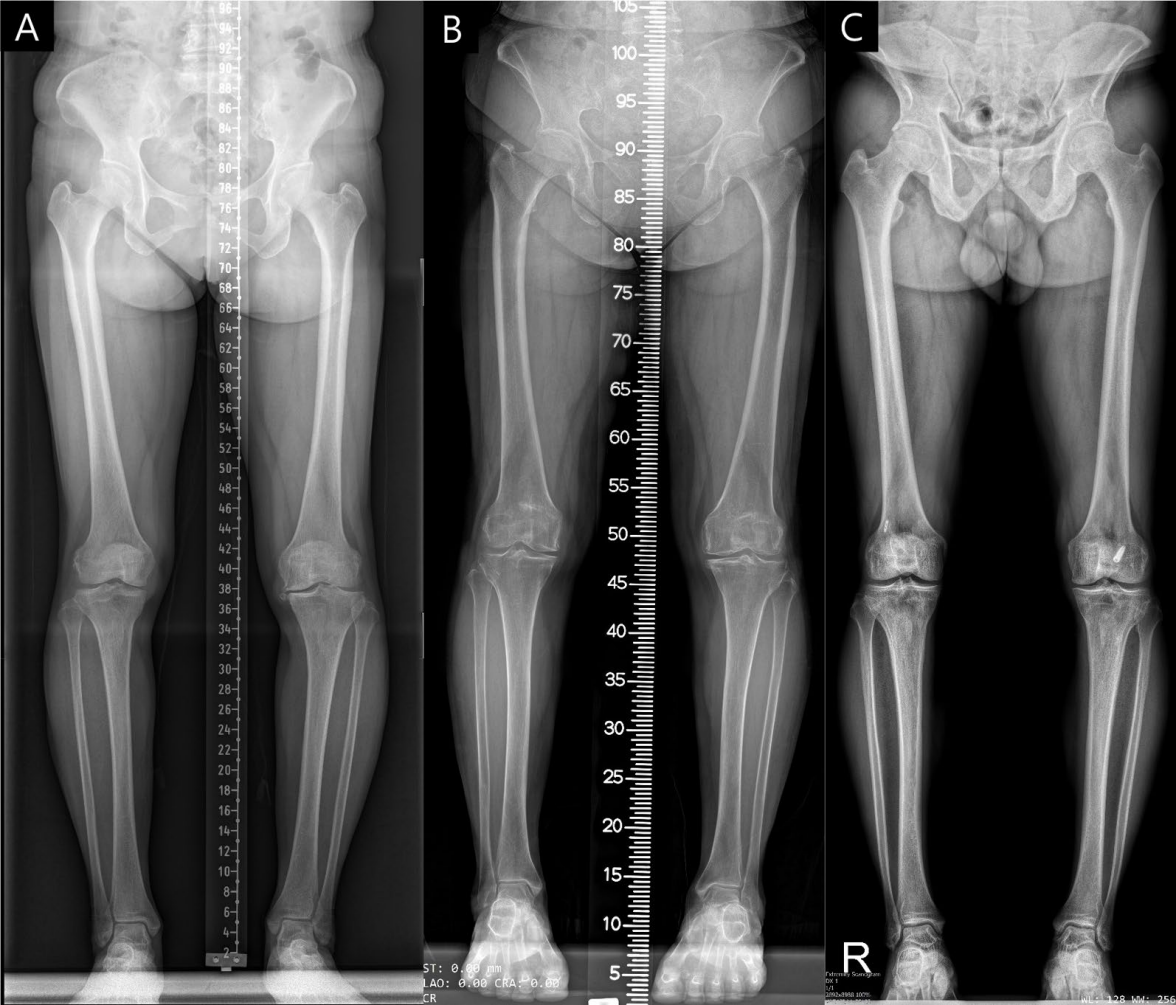
Examples of full-leg radiographs from Institutes **A**, **B**, and **C**, with each letter representing the corresponding institute

The imaging devices and protocols across Institutes A, B, and C differed. Besides differences in image resolution and contrast, Institutes A and B generated full-leg radiographs by merging separate images of the hip, knee, and ankle, each incorporating a different type of ruler. Meanwhile, Institute C employed a biplanar X-ray imaging system (EOS) to capture the entire leg in one shot, without including a ruler in the image (Fig. 2).

The model's accuracy was assessed using the inter-observer intraclass correlation coefficient (ICC), comparing DL-generated measurements of radiologic parameters with ground-truth annotations from the orthopedic specialist. To evaluate the model’s consistency, intra-observer ICCs were compared between the orthopedic specialist and the DL model. For this intra-observer ICC assessment, measurements were repeated 4 weeks after the initial evaluation. Bland–Altman plots were plotted to visualize measurement differences and outliers. We also measured the percentage of absolute errors over 1.5 degrees from the measurement of the orthopedic specialist [5]. Additionally, the performance of the application programming interface (API) was assessed by measuring the time elapsed during evaluation.

### Statistical analysis

All statistical analyses were conducted using Python 3.10.12. Nominal data were expressed as percentages and analyzed using either Pearson's chi-square test or Fisher's exact test. Continuous data were presented as means ± standard deviations (SD) and analyzed using the Student's t-test. A p-value below 0.05 was considered statistically significant.

## Results

**Fig. 3:**
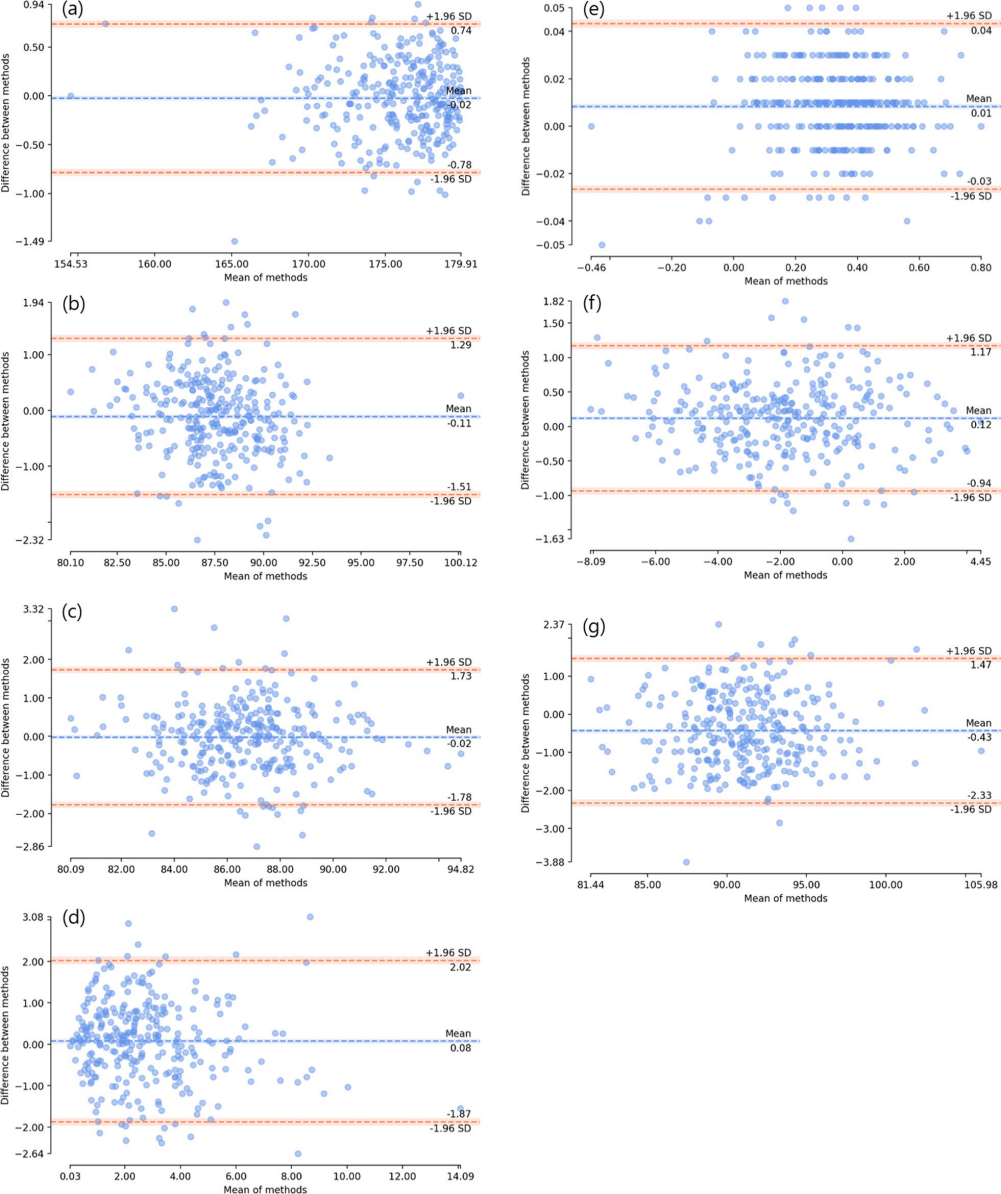
Bland–Altman plots of the total validation set for each radiologic parameters. **a** hip-knee-ankle angle. **b** lateral distal femoral angle. **c** medial proximal tibial angle. **d** joint line convergence angle. **e** weight-bearing line ratio. **f** joint line obliquity angle. **g** lateral distal tibial angle

Patient demographics of the training set are presented in Table 1. Inter-observer ICC with 95% confidence intervals (CI), for radiologic parameters by institute are shown in Table 2. These parameters have a total inter-observer ICC ranging from 0.936 to 0.997, indicating excellent accuracy. Table 3 presents the intra-observer ICC for these parameters when compared with the evaluations of the orthopedic specialist. Figure 3 illustrates the Bland–Altman plots for all 300 radiographs, categorized by their respective radiologic parameters. The results demonstrated that there was no significant systemic variation, with 95% CI for all angles including zero degrees of difference.

**Table 1:** Demographic characteristics of the training set

*N* = 11,212	Mean (SD)
Age (years)	62.7 (13.0)
Sex (%)	
Female	73.8%
Male	26.2%
Height (cm)	157.7 (8.9)
Weight (kg)	63.7 (10.8)
Body mass index (kg/m^2^)	25.6 (3.6)

SD, standard deviation

**Table 2:** Inter-observer reliability of the radiologic parameters according to different institutes: Comparison of the deep learning model measurements with an orthopedic specialist (ground-truth)

	ICC (Orthopedic specialist, Ground-truth)	ICC (Institute A)	ICC (Institute B)	ICC (Institute C)	ICC (Total)
HKAA	1.00 (1.00–1.00)	0.995 (0.99–1.00)	0.998 (1.00–1.00)	0.994 (0.99–1.00)	0.997 (1.00–1.00)
LDFA	1.00 (1.00–1.00)	0.969 (0.93–0.98)	0.976 (0.96–0.98)	0.982 (0.97–0.99)	0.976 (0.97–0.98)
MPTA	1.00 (1.00–1.00)	0.966 (0.95–0.98)	0.962 (0.94–0.97)	0.956 (0.93–0.97)	0.963 (0.95–0.97)
JLCA	1.00 (1.00–1.00)	0.892 (0.84–0.93)	0.950 (0.93–0.97)	0.913 (0.87–0.94)	0.936 (0.92–0.95)
WBL ratio	1.00 (1.00–1.00)	0.993 (0.91–1.0)	0.998 (1.00–1.00)	0.997 (1.00–1.00)	0.987 (0.98–0.99)
JLOA	1.00 (1.00–1.00)	0.986 (0.98–0.99)	0.979 (0.97–0.99)	0.989 (0.98–0.99)	0.987 (0.98–0.99)
LDTA	1.00 (1.00–1.00)	0.979 (0.91–0.99)	0.977 (0.97–0.99)	0.977 (0.96–0.99)	0.979 (0.96–0.99)

*ICC* intraclass correlation coefficient, *HKAA* hip-knee-ankle angle, *LDFA* lateral distal femoral angle, *MPTA* medial proximal tibial angle, *JLCA* joint line convergence angle, *WBL* weight bearing line, *JLOA* joint line obliquity angle, *LDTA* lateral distal tibial angle

**Table 3:** Intra-observer intraclass correlation coefficient (ICC) of the radiologic parameters

	ICC (Current model)	ICC (Orthopedic specialist)
HKAA	1.00 (1.00–1.00)	0.997 (0.99–1.00)
LDFA	1.00 (1.00–1.00)	0.987 (0.98–0.99)
MPTA	1.00 (1.00–1.00)	0.969 (0.96–0.98)
JLCA	1.00 (1.00–1.00)	0.950 (0.93–0.96)
WBL ratio	1.00 (1.00–1.00)	0.997 (0.97–1.00)
JLOA	1.00 (1.00–1.00)	0.991 (0.99–0.99)
LDTA	1.00 (1.00–1.00)	0.986 (0.98–0.99)

*HKAA* hip-knee-ankle angle, *LDFA* lateral distal femoral angle, *MPTA* medial proximal tibial angle, *JLCA* joint line convergence angle, *WBL* weight bearing line, *JLOA* joint line obliquity angle, *LDTA* lateral distal tibial angle

Table 4 provides a comparison of the percentage of absolute errors greater than 1.5 degrees against the evaluations of the orthopedic specialist. Notably, the HKAA consistently demonstrated an absolute error below 1.5 degrees (0%). In contrast, the JLCA and LDTA had a higher propensity for errors beyond this threshold across all institutions: JLCA varied between 13.6% and 21.9%, whereas LDTA ranged from 16.3% to 28.2%.

**Table 4:** Percentage of absolute error over 1.5 degrees compared to ground-truth (orthopedic specialist)

	Absolute error (Institute A)	Absolute error (Institute B)	Absolute error (Institute C)	Absolute error (Total)
HKAA	0	0	0	0
LDFA	4.2	5.3	3.1	4.2
MPTA	7.5	14.9	9.9	10.7
JLCA	13.6	21.9	14.9	16.7
WBL ratio*	N/A	N/A	N/A	N/A
JLOA	1.0	2.0	1.0	1.4
LDTA	28.2	16.3	19.1	20.1

All values are in percentages

*Absolute error of 1.5 degrees was not applied to WBL ratio as it is a ratio

*HKAA* hip-knee-ankle angle, *LDFA* lateral distal femoral angle, *MPTA* medial proximal tibial angle, *JLCA* joint line convergence angle, *WBL* weight bearing line, *JLOA* joint line obliquity angle, *LDTA* lateral distal tibial angle, *N/A* not assessed

Lastly, a substantial reduction in processing time with our enhanced model was achieved. Whereas the prior model took approximately 10–11 s, our updated version shortened this time by over 30-fold to an average of just 300 ms.

## Discussion

The most important finding of this study is the consistent reliability of our enhanced DL model in analyzing radiographs across different institutions, each with varying X-ray equipment, while maintaining high accuracy. Furthermore, the introduction of additional radiologic parameters not only broadened the model's scope but also demonstrated a high degree of precision. Impressively, despite the incorporation of these additional parameters, the time required for measurements was significantly reduced.

Recent studies in coronal limb alignment demonstrate ongoing enhancements in DL models, now comparable to human specialist performance [4–9]. For instance, Gielis et al. implemented a random forest regression model for measuring HKAA, focusing on the femur and tibia outlines [18]. Nguyen et al. adopted a two-stage method, initially extracting required ROIs followed by CNN-based exact required point detection [9]. Tack et al. combined YoLOv4 and Resnet Landmark Regression Algorithm (YARLA) for comprehensive full-leg radiograph analysis [5], while Erne et al. and Jo et al. developed DL models for limb alignment assessment, even with embedded implants [6, 7].

With the increasing integration of DL algorithms in radiographic analyses, it is necessary to validate the applicability of such developed models within a generalized population. A key concern is the dependence on retrospective data during model training, which can introduce selection bias, particularly when the training dataset is small [19, 20]. In addition, the performance of models in previous studies often lacks validation on radiographs from other institutes with different imaging devices [6]. For instance, a robust model might fail to accurately recognize anatomic landmarks in radiographs from different institutes, underscoring the importance of external validation. In this study, our DL model was trained on a set comprising over 10,000 radiographs, implying broad applicability. However, given previous findings of decreased algorithm performance on external datasets [21], we externally validated its performance on other datasets and observed excellent results. This success can be attributed to the model's segmented approach: rather than conducting a holistic landmark detection, it distinctly identifies and assesses ROIs for the hip, knee, and ankle, which are then integrated. Consequently, this methodology guarantees consistent accuracy in measurements, even amid variances in equipment or protocols across different institutes.

The inclusion of parameters such as WBL ratio, JLOA, and LDTA expands the applicability of a single full-leg radiograph to various purposes. In addition, it may potentially reduce the need for additional X-rays targeting specific regions, thereby reducing cost and radiation exposure. The WBL ratio is a prevalent metric for determining alignment. It also acts as a surrogate marker, indicating the primary pathway of weight, and is crucial in performing osteotomy. JLOA, representing knee joint line orientation, is utilized to assess outcomes associated with knee osteotomies. Meanwhile, LDTA is a measure of ankle alignment and holds significance not only in determining alignment but also in various ankle surgeries.

In evaluating HKAA, the DL model performed exceptionally, recording no instances of absolute error surpassing 1.5 degrees. In contrast, the JLCA and LDTA measurements had a higher incidence of such errors. This finding is consistent with previous research: Gielis et al. reported an inter-observer ICC for HKAA ranging from 0.82 to 0.90 [18]. Na et al. reported an inter-observer ICC for JLCA between 0.73 and 0.82 [22], while Hodel et al. observed an inter-observer ICC of 0.79 for LDTA [23]. These earlier findings align with our results, which highlight the pronounced reliability and low error associated with HKAA in contrast to the slightly diminished reliability and elevated error seen in JLCA and LDTA.

One potential reason for the elevated error rate in JLCA could be its more localized focus. Due to its concentrated measurement area, JLCA is more susceptible to minor landmark positioning errors compared to HKAA, which covers the entire lower extremities. Meanwhile, the increased error rate in LDTA may be attributed to its reliance on only three landmarks for ankle identification, as opposed to the eleven designated for the knee. Furthermore, the DL model faces challenges when the vectors at the prediction point share substantial prior information with other vectors, leading to minor fluctuations in accuracy. This issue is particularly evident in the regions inferred for JLCA and LDTA, such as the femoral epicondyles, tibial plateau, and tibial plafond, where predictive difficulties arise due to vector similarities. In the case of LDTA, although the ankle joint is clearly visible to the naked eye, the AI model tends to find high similarity with surrounding vectors, resulting in less stable predictions. Notwithstanding these errors, our model's measurements closely matched those of the orthopedic specialist, consistently outperforming previous research by demonstrating superior inter-observer ICC values across all parameters.

In this updated model, the enhanced speed coupled with sustained high accuracy benefits not only medical practitioners but also patients and researchers. This can be attributed to the utilization of DEKR [16]. In the previous model, U-Net was employed for model implementation. U-Net's characteristic feature is its use of contracting and expanding paths to extract feature maps, enabling consideration of both low-level and high-level information simultaneously [24]. Although this down-scale to up-scale approach showed high performance in segmentation tasks, it exhibited weaknesses in keypoint detection (Heatmap), which demands precise inference within a narrow range.

To overcome this limitation, we adopted the High-resolution-Net (HR-Net) based DEKR, which incorporates a strategy of iterative multi-branching at different low-level and high-level dimensions [25]. HR-Net, known for achieving state-of-the-art performance in pose estimation with narrow inference ranges, preserves high resolution throughout the process through parallel connections of high-to-low resolution [26]. This ensures the retention of fine details in Heatmaps without the need for low-to-high processes. Additionally, DEKR employs a multi-scale fusion approach to repeatedly support low-resolution representations at the same depth and similar levels as high-resolution representations, resulting in improved accuracy in narrower range inference for Heatmaps [27]. Furthermore, the Non-Maximum Suppression technique described in DEKR allows for obtaining accurate keypoint candidates, further enhancing the overall performance when compared to the previous model based on U-Net [28, 29].

The utilization of this DL model offers promising applications in the clinical domain. Its ability to rapidly and accurately analyze alignment is particularly beneficial in fast-paced clinical environments, providing essential information to both clinicians and patients. In addition, the model significantly streamlines the manual measurement process of large volumes of radiographs for research purposes. Researchers can upload radiographs into the DL model and engage in other tasks while the model autonomously analyzes the extensive data. This capability allows for a considerable expansion in research scope, enabling the exploration of more diverse and larger patient populations. Thus, the DL model represents a valuable tool for enhancing both clinical efficiency and research productivity.

The study is not without limitations. First, the training process, specifically the landmark labeling, was conducted manually and requires a high level of accuracy and precision. Also, the model may be susceptible to outliers in radiographs that deviate from the distribution of the training dataset, making the localization of landmarks challenging and potentially leading to a decrease in accuracy. While the inter-observer reliability across all radiologic parameters surpasses previous studies, there remains a notable percentage of absolute errors exceeding 1.5 degrees in JLCA and LDTA, underscoring a need for refinement. Furthermore, our model does not currently differentiate mal-rotated full-leg radiographs, meaning it cannot assess the quality of a true antero-posterior view. Such inaccuracies in radiographs can lead to measurement discrepancies in radiologic parameters. Future enhancements of the model could focus on assessing rotational errors and improving the detection of suboptimal beam projection angles to achieve a true antero-posterior view. Additionally, the validation set did not categorize radiographs based on osteoarthritis severity or knee deformity grade, which could affect model performance with severely pathological cases. However, it is noteworthy that in clinical practice, measurements are often required on complex radiographs, and our model demonstrated satisfactory performance even when these challenging images were included.

## Conclusions

The enhanced DL model demonstrated a universal applicability across various full-leg radiographs from different institutions, exhibiting accuracy and reliability on par with an orthopedic specialist. Our model performed a number of measurements in the full-leg radiographs, with accuracy and reliability comparable to that of the orthopedic specialist. The model’s precision and expedited processing time hold promise for both clinical settings and research endeavors involving large volumes of radiographs.

## Data Availability

The datasets used and/or analysed during the current study are available from the corresponding author on reasonable request.
